# Influence of a neck compression collar on cerebrovascular and autonomic function in men and women

**DOI:** 10.1371/journal.pone.0225868

**Published:** 2019-12-02

**Authors:** Hitesh Joshi, Loriann M. Hynes, Heather Edgell

**Affiliations:** 1 School of Kinesiology and Health Sciences, York University, Toronto, ON, Canada; 2 Muscle Health Research Centre, York University, Toronto, ON, Canada; University of Massachusetts Boston, UNITED STATES

## Abstract

**Objective:**

Neck compression collars have been proposed to reduce injury to the brain caused by head impacts. Our objective was to test if compression of the carotid artery affected the baroreflex and influenced blood pressure control.

**Methods:**

Cerebrovascular and autonomic responses of healthy young men and women (n = 8 each) to paced deep breathing, Valsalva, and 70^o^ head-up tilt with or without use of a Q-collar were determined. Continuous measurements of heart rate, beat-to-beat blood pressure, transcranial Doppler, and end-tidal gases were obtained. Heart rate variability was measured during supine rest and head-up tilt. Carotid artery and jugular vein cross-sectional area were measured at end-inhalation and end-exhalation using cross-sectional ultrasound images at diastole.

**Results:**

Wearing the collar reduced carotid cross-sectional area (CSA; P = 0.022; η^2^ = 0.03) and increased jugular CSA (P = 0.001; η ^2^ = 0.30). In both men and women, wearing the collar increased systolic blood pressure during Valsalva (P<0.05; η ^2^ = 0.38). In only men, wearing the collar resulted in prolonged pressure recovery time during Valsalva (P = 0.02; η ^2^ = 0.05). In only women, wearing the collar increased baseline diastolic (P = 0.026; η ^2^ = 0.09) and mean (P = 0.041; η ^2^ = 0.06) middle cerebral artery (MCA) blood flow velocity, which attenuated the normal increase of diastolic (P = 0.01; η ^2^ = 0.03) and mean (P = 0.038; η ^2^ = 0.02) MCA blood flow velocity during Valsalva. There were no effects of sex or collar on the responses to deep breathing (P>0.05), and there were no effects of the collar on cerebrovascular function, hemodynamics, cardiovagal baroreceptor sensitivity, or heart rate variability (P>0.05) during upright tilt.

**Conclusion:**

Use of the Q-collar compresses both the jugular vein and carotid artery influencing sympathetic nerve activity in both men and women while influencing brain blood flow in women.

## Introduction

The use of jugular compression collars (Q-collar) during physical activity has recently been shown to reduce white matter alterations associated with head impact exposure in male and female athletes [1–4]. These changes are proposed to be due to increased cerebral blood volume since jugular vein compression has been shown to increase both cerebral blood flow [5, 6] and intracranial venous volume [7], and the use of rigid neck collars has also been shown to increase intracranial pressure [8]. However, the jugular vein lies in close proximity to the carotid artery and carotid sinus wherein lie the arterial baroreceptors. Many previous studies have found that neck collar pressure elicits an increase of sympathetic activity and blood pressure due to the reduced transmural pressure across the artery [8–11]. While the use of the Q-collar may be beneficial for its role in reducing injury to the brain caused by head impacts [1–3], its use has not yet been investigated for its potential influence on autonomic function or the cerebrovascular response to changes in blood pressure.

The greater sympathetic activity and/or increased cerebral volume elicited by neck pressure could be beneficial for individuals with orthostatic intolerance by maintaining or increasing cerebral perfusion pressure in an upright posture. Women have a greater prevalence of orthostatic intolerance compared to men [12] even though women have been shown to have similar sympathetic baroreflex sensitivity during a Valsalva maneuver and similar sympathetic activity during head up tilt [13]. However, women have been shown to have attenuated cerebrovascular resistance during upright posture and a greater increase of diastolic and mean cerebral blood flow velocity during Valsalva compared to men [14] implying sexually dimorphic changes in dilation/constriction of the cerebrovasculature to maintain flow. Indeed, sex differences in cerebral autoregulation have recently been described, however, studies have found conflicting results [15, 16].

The purpose of this study was to investigate if the Q-collar affected the baroreflex and/or brain blood flow in healthy, young men and women. We hypothesized that using a Q-collar would not only compress the jugular vein, but would also compress the carotid artery leading to baroreceptor inhibition and an increase of sympathetic control of blood pressure. We further hypothesized that these changes would lead to less hypotension and greater brain blood flow during tilt, particularly in women.

## Materials and methods

All procedures were approved by the Research Ethics Board of York University, and written informed consent was provided by all participants. Each participant acknowledged that they were not to be identified and we have fully anonymized them. We complied with all mandatory laboratory health and safety procedures when conducting these experiments. All procedures performed were in accordance with the ethical standards of the institutional research committee and with the latest revision of the Declaration of Helsinki.

### Participants

Young men (n = 8, 22±3years, 178±11cm, 77±14kg) and women (n = 8, 22±4years, 161±6cm, 60±9kg) were recruited to participate and did not have previously diagnosed cardiovascular disease. However, one male participant was found to have hypertension at the beginning of testing (141/84), and another male participant exhibited hypertension as testing progressed (progression from 115/56 before any tests to 157/72 at the beginning of the 1^st^ Valsalva trial). Women were not taking oral contraceptives and were tested between days 2–5 of the menstrual cycle (early follicular phase) when sex hormones are at minimal levels. All participants were asked to refrain from smoking (none were habitual smokers), caffeine, heavy exercise, and eating fatty foods 12 hours prior to testing (participants were not fasted).

### Cardiovascular and respiratory measurements

Continuous blood pressure was determined using finger photoplethysmography (NexFin BMEye, Netherlands), and was calibrated to an automated measurement (BPTru Medical Devices, Canada) which was taken before testing. The finger cuff of the NexFin was maintained at heart level at all times. Heart rate (HR) was measured with a standard single lead electrocardiogram. Stroke volume (SV) was obtained using the Modelflow algorithm from the NexFin, and cardiac output (Q) was determined by multiplying SV and heart rate. Stroke volume index (SVi) and cardiac output index (Qi) were normalized to body surface area (Dubois and Dubois formula). Total peripheral resistance index (TPRi) was calculated as mean arterial pressure (MAP)/Qi. End-tidal CO_2_ and O_2_ were sampled using a nasal cannula connected to gas analyzers (Vacumed, USA). End-tidal gases were not obtained for 2 male participants due to displacement of the nasal cannula during testing. Respiratory rate was determined from the breath-to-breath peaks in end-tidal CO_2_.

Brain blood flow velocity through the middle cerebral artery (MCA) was measured with transcranial Doppler (TCD; Multigon Industries Inc., USA). A 2-MHz TCD probe was placed on the right side of the head in the temporal window and held in place by an adjustable headband. Cerebral perfusion pressure was calculated as MAP—0.7355mmHg/cmH_2_O * distance from the transcranial Doppler probe to the heart. Cerebrovascular Resistance Index (CVRi) was calculated as cerebral perfusion pressure/mean MCA velocity. Resistance index (RI) was calculated as RI = (MCA systolic–MCA diastolic)/MCA systolic. Pulsatility index (PI) was calculated as PI = (MCA systolic -MCA diastolic)/MCA mean.

Cross-sectional ultrasound images (Vivid i, GE Healthcare, Canada) of the carotid artery and jugular vein were taken above the collar using a 9L-RS linear transducer (3.0–10.0MHz) following the autonomic testing protocols with participants in a seated position. Images were first taken ~5minutes after placement of the Q-collar, and a mark was made to indicate where the probe was positioned. The collar was then removed and images were taken again at the marked probe location and using anatomical landmarks such as the sternocleidomastoid muscle. EchoPAC ultrasound software (GE Healthcare, Canada) was used to measure the cross-sectional area of the carotid artery and jugular vein (cm^2^). In an attempt to control for breathing and its effects on jugular and carotid flow, participants were asked to take a deep breath in, hold for 3 seconds (without a closure of the glottis) and a short video loop was taken. Participants were then asked to return to normal breathing for approximately a minute before a deep exhalation which was maintained for 3 seconds (without closure of the glottis). Another short video loop was taken at this time. Cross-sectional area of both the artery and vein were determined at diastole.

### Protocol

Q-collars (provided by Q30 Innovations) were sized according to Q30 Innovation guidelines. The collars sat at a comfortable position for the participants at the base of the neck with the opening at the front. Participants completed the testing protocols described below twice, with or without use of a Q-collar (randomized). After placement of the equipment (~5minutes), participants underwent 1) 5 minutes of supine rest, 2) paced deep breathing of 6 breaths/min (5s inhale, 5s exhale; 8 cycles) in duplicate, 3) 15s Valsalva maneuver to 40mmHg mouth pressure in duplicate, and 4) 5 minutes of baseline measurements followed by 10 minutes of 70^o^ head-up tilt. One minute prior to the Valsalva maneuver, participants were tilted to a 20^o^ head-up position to prevent a flat-top response[17], and visual feedback was provided to participants for their achieved pressure. All trials were separated by a minimum of 5 minutes of supine rest, and the collar/no collar trials were separated by a minimum of 10 minutes of supine rest.

### Data analysis

All signals were obtained using a Powerlab data acquisition device and LabChart Pro software (ADInstruments, USA). For the deep breathing trials, maximum and minimum heart rate was determined from 6 breath cycles. The change in HR and the ratio between maximum and minimum (Exhalation:Inhalation ratio; E:I ratio) were calculated. Thirty second averages for systolic, diastolic, and mean blood pressure and brain blood flow velocity were determined prior to the Valsalva maneuver, and values were also determined at the minimum of Phase 2 (i.e. the minimum blood pressure during forced exhalation due to reduced venous return/stroke volume), at the end of Phase 2 (i.e. the maximum blood pressure at the end of forced exhalation due to compensatory sympathetic/adrenergic activation), and at Phase 3 (i.e. the minimum blood pressure immediately after forced exhalation due to reperfusion of pulmonary vessels and lower stroke volume). Pressure recovery time (PRT) was determined as the time from Phase 3 to the time at which systolic blood pressure returned to baseline. Adrenergic score (BRSa) was calculated using systolic blood pressure according to Novak [18] as ((Baseline-Phase2min)+(Phase2end-Phase2min)*0.7))/PRT and according to Low [19] as ((Baseline-Phase2min)+0.75*(Phase2end-Phase3))/PRT. The Valsalva HR ratio was determined as the ratio between the maximum and minimum heart rates after Phase 3 of the Valsalva (i.e. HR increases to a maximum during Phase 2 and Phase 3 due to lower stroke volume; however, HR decreases abruptly after forced exhalation ends due to parasympathetic influence of the baroreceptor in order to reduce blood pressure to a homeostatic level, compensating for the sympathetic/adrenergic activation of Phase 2). The changes in systolic, diastolic, and mean blood pressure and brain blood flow velocity were calculated as Phase2min-Baseline and Phase2end-Baseline. One minute averages for all cerebrovascular, hemodynamic, and respiratory data were calculated one minute prior to upright tilt, and during the last minute of upright tilt. Heart rate variability (HRV; LabChart Pro 8.0, ADInstruments, USA) was determined from the ECG recording for 5 minutes prior to tilt and for the last 5 minutes of tilt. A Hann (cosine-bell) data window was used with a window overlap of 50%. Fast Fourier transform size was 1024. The low-frequency spectrum (LF) was 0.04–0.15Hz and the high frequency (HF) spectrum was 0.15–0.45Hz). Cardiovagal baroreceptor sensitivity was determined with the spontaneous method[20, 21] at the same time points as HRV.

**Fig 1:**
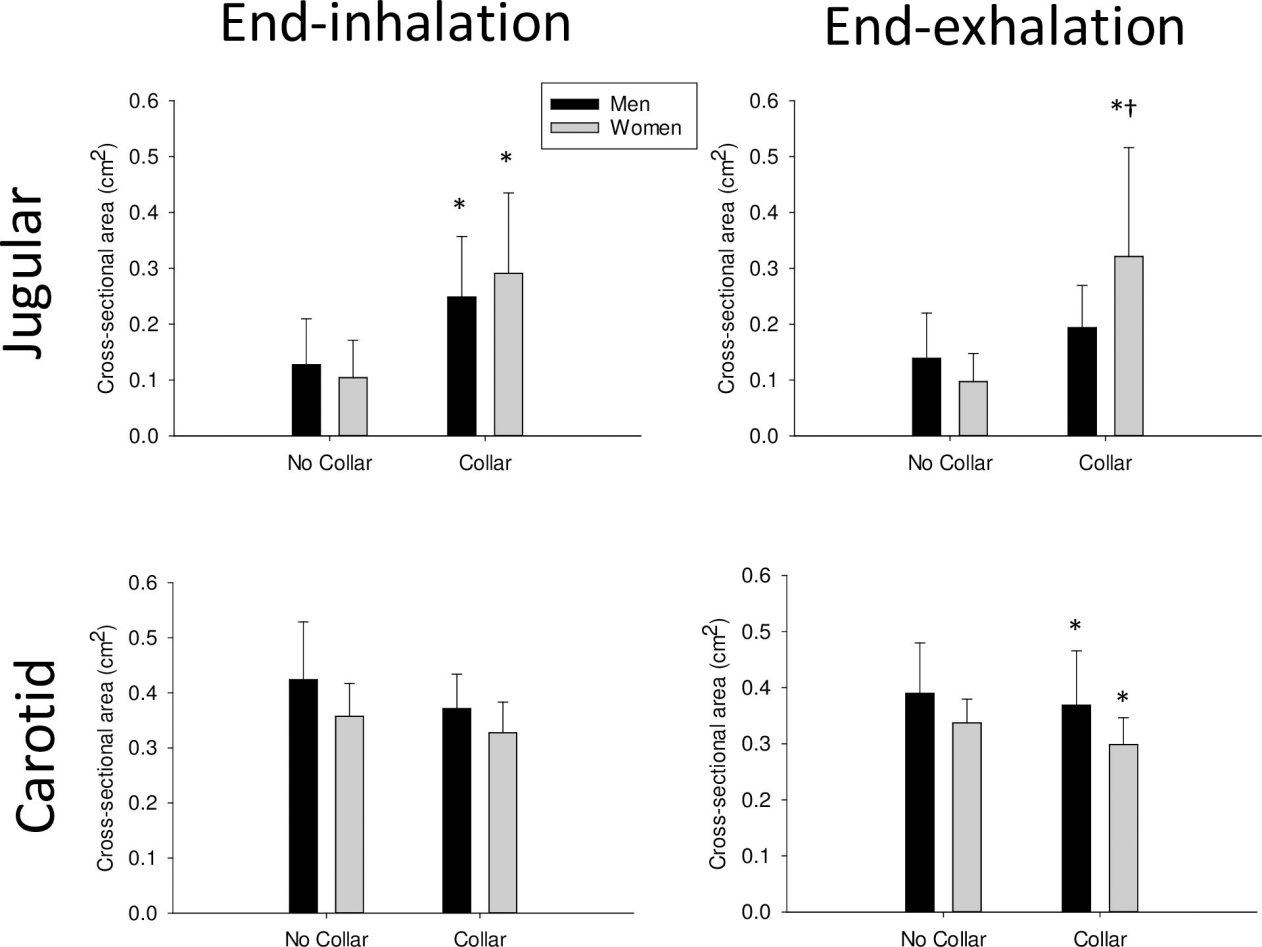
**Jugular vein (top panels) and carotid artery (bottom panels) cross sectional area at the end of an inhalation (left panels) and at the end of an exhalation (right panels) in men and women with or without use of a compression collar.** Men are black bars, women are grey bars. *indicates a significant effect of Collar; †indicates a significant effect of sex within group; values are mean ± 95% Confidence Interval.

**Fig 2:**
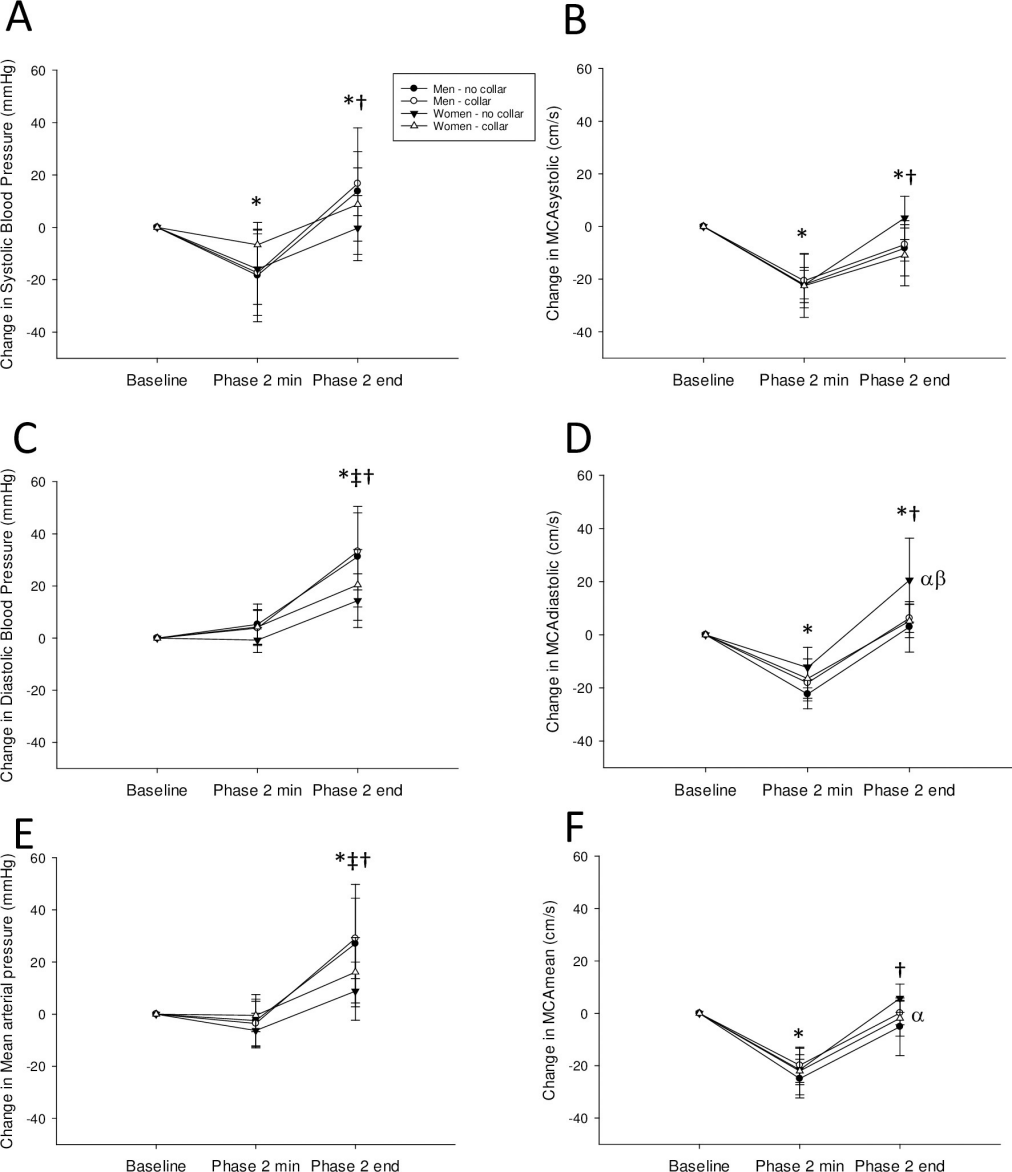
**Change in systolic blood pressure (A), systolic middle cerebral artery (MCA) velocity (B), diastolic blood pressure (C), diastolic MCA velocity (D), mean arterial pressure (E), and mean MCA velocity (F) from baseline, to the Phase 2 minimum during a Valsalva maneuver, and to the end of Phase 2 of the Valsalva maneuver.** Men are circles, women are triangles, without the collar are black symbols, with the collar are white symbols. *indicates significant from Baseline, †indicates significant from Phase 2 min; ‡indicates a sex difference at that timepoint; α indicates a sex difference between No collar groups; β indicates a Collar effect within group; values are mean ± 95% Confidence Interval.

Jugular vein and carotid artery cross-sectional area (Fig 1), HR responses to deep breathing (Table 1), adrenergic responses to Valsalva (Table 2), and the maximum HR response to tilt were analyzed with two-way repeated measures ANOVAs with one repeated factor (collar use) and one between group factor (sex; Sigmaplot 13.0). The cBRS, HRV, and cardiorespiratory responses to tilt (Table 3) were analyzed with three-way ANOVA (sex, collar, and tilt as factors). The change in blood pressure and brain blood flow velocity over time during the Valsalva maneuver (Fig 2) were also analyzed with three-way ANOVA (sex, collar, and time as factors). Effect size (η ^2^) was determined as the Sum of Squares (effect)/Sum of Squares (total). Data in figures and tables are presented as mean ± 95% confidence interval. Significance was set at P<0.05.

**Table 1:** Heart rate responses to paced deep breathing with or without use of jugular compression collar.

		Maximum HR (bpm)	Minimum HR (bpm)	Change in HR (bpm)	E:I ratio
Men	No Collar	81.3±10.2	56.2±5.2	25.1±7.6	1.45±0.13
	Collar	80.9±12.0	54.3±5.0	26.6±7.7	1.48±0.11
Women	No Collar	80.6±6.1	58.8±6.3	21.8±8.0	1.39±0.17
	Collar	74.9±8.7	56.3±7.9	18.6±7.6	1.35±0.17

HR is heart rate; E:I ratio is the ratio of HR response between inhalation and exhalation; Values are mean ± 95% Confidence Interval

**Table 2:** Baseline hemodynamic and MCA velocity values and adrenergic responses to Valsalva.

	Male		Female	
	No Collar	Collar	No Collar	Collar
Baseline SBP (mmHg)†	136.5±10.4	137.7±12.1	107.8±7.7	107.0±2.9
Baseline DBP (mmHg)	74.8±7.9	75.2±7.9	70.8±8.8	72.6±6.6
Baseline MAP (mmHg)	90.5±7.2	91.2±7.3	81.8±7.3	83.5±5.3
Baseline MCAsystolic (cm/s)	94.7±16.6	94.9±15.0	90.9±7.5	101.2±15.6
Baseline MCAdiastolic (cm/s)	40.9±8.7	37.8±9.2	33.7±9.1	42.6±7.3*
Baseline MCAmean (cm/s)	60.3±11.4	56.8±10.0	58.2±5.3	65.3±8.3*
Baseline HR (bpm)	68.4±7.1	66.7±7.7	70.1±6.3	69.1±5.6
Phase 2 min SBP (mmHg)†	114.6±12.4	117.8±10.0*	92.4±12.6	99.3±9.6*
Phase 2 end SBP (mmHg)†	149.9±27.1	153.3±22.3*	107.4±13.8	115.7±15.4*
Phase 3 SBP (mmHg)	119.9±30.2	119.1±19.5	97.5±11.8	103.0±11.1
Baseline—Phase 2min (SBP; mmHg)	22.0±17.6	15.4±18.3*	14.5±12.7	8.5±9.1*
Phase 2end—Phase 2min (SBP; mmHg)	35.2±26.4	35.5±25.1	14.9±13.8	16.3±9.4
Phase 3 –Phase 2end (SBP; mmHg)†	-30.0±8.8	-34.2±9.7	-9.9±6.6	-12.7±11.1
PRT (s)	4.9±1.9	6.4±0.9*	5.8±1.1	5.6±1.8
BRSa (Novak)	11.1±6.1	6.3±5.3	3.9±2.9	4.0±2.5
BRSa (Low)†	13.5±10.4	6.5±2.9	3.7±1.4	3.9±2.7
Maximum HR (bpm)	114.6±8.6	112.2±8.2	118.0±14.0	114.0±10.8
Minimum HR (bpm)	52.4±4.2	54.7±6.8	53.5±5.1	52.9±4.2
Valsalva HR Ratio	2.2±0.2	2.1±0.2	2.2±0.3	2.2±0.3

SBP is systolic blood pressure; DBP is diastolic blood pressure; MAP is mean arterial blood pressure; BRSa is adrenergic score

†indicates a significant effect of sex

*indicates a significant effect of collar; SBP is systolic blood pressure; PRT is pressure recovery time; BRSa is adrenergic baroreflex sensitivity; HR is heart rate; values are mean ± 95% Confidence Interval

**Table 3:** Cardiorespiratory and heart rate variability responses to 10 minutes of 70o upright tilt with or without the use of Q-collar.

	Men				Women			
	No Collar		Collar		No Collar		Collar	
	Baseline	Tilt	Baseline	Tilt	Baseline	Tilt	Baseline	Tilt
cBRS (ms/mmHg)†	22.3±11.6	6.7±1.9*	20.4±7.6	6.4±2.5*	55.2±19.8	17.6±9.1*	48.6±19.9	20.9±7.5*
SDNN (ms)	65.8±17.4	48.7±13.0*	81.1±26.4	54.5±19.5*	67.3±34.5	46.1±9.6*	64.2±25.7	53.3±25.0*
RMSSD (ms)	50.9±19.3	21.4±6.3*	56.8±24.4	27.8±15.3*	54.2±27.6	22.7±6.9*	49.5±22.1	26.0±131*
pRR50 (%)	28.2±17.2	4.0±3.2*	31.3±16.5	6.6±6.1*	25.8±19.1	3.6±5.4*	22.1±17.1	7.3±10.7*
Total Power (μs^2^)	5010±2878	2204±978*	7968±5435	3133±1856*	4826±4618	2461±1168*	5377±4992	2980±2271*
LF (nu)	51.3±15.9	85.6±8.9*	58.2±10.4	79.9±14.5*	47.3±10.1	79.6±9.9*	55.2±17.5	78.7±9.0*
HF (nu)	47.5±15.7	15.3±9.2*	40.4±11.0	20.7±14.5*	51.7±10.0	21.4±9.8*	43.6±16.7	21.9±8.5*
LF/HF	1.53±1.15	8.62±4.44*	1.70±0.81	8.84±6.2*	1.01±0.41	4.93±2.30*	1.99±1.71	5.56±4.95*
SD1 (ms)	36.0±13.7	15.1±4.5*	40.2±17.3	19.7±10.9*	38.4±19.6	16.1±4.9*	35.1±15.6	18.4±9.3*
SD2 (ms)	84.9±23.5	67.1±18.1*	106.7±34.7	74.2±26.0*	86.9±44.9	62.9±13.4*	83.3±33.7	72.9±34.2*
Heart rate (bpm)	63.5±7.2	86.5±8.5*	62.4±7.1	78.3±10.2*	63.8±5.3	83.7±9.8*	65.9±6.9	83.6±9.5*
SBP (mmHg)†	136.3±14.5	128.4±15.8	135.0±6.1	132.0±8.2	106.9±9.7	102.0±11.8	109.0±7.7	106.3±10.7
DBP (mmHg)	74.2±5.0	78.4±5.4	72.8±8.0	79.1±5.0	71.4±10.2	71.8±9.3	76.7±7.9	77.1±8.0
MAP (mmHg)†	90.5±5.0	92.6±6.3	89.6±7.3	94.5±4.4	82.4±8.6	81.2±9.0	87.4±7.4	86.6±8.2
CPP (mmHg)†	90.5±5.0	76.5±6.2*	89.6±7.3	78.4±4.4*	82.4±8.6	69.5±11.2*	87.4±7.4	72.9±8.6*
MCA mean (cm/s)†	59.8±9.4	52.4±10.4*	58.0±9.8	50.4±7.9*	66.8±7.2	56.2±7.1*	66.8±7.6	53.5±6.6*
MCA systolic (cm/s)	96.1±14.9	84.5±16.4*	96.1±13.9	84.0±11.0*	100.9±12.2	87.6±10.2*	102.6±15.8	90.2±9.6*
MCA diastolic (cm/s)	40.4±6.8	36.1±8.0*	38.5±7.8	33.8±7.3*	44.3±6.1	38.6±7.1*	43.4±7.5	32.4±9.6*
Resistance index	0.58±0.03	0.57±0.04	0.60±0.04	0.60±0.06	0.56±0.07	0.56±0.07	0.57±0.09	0.63±0.11
Pulsatility index	0.93±0.06	0.93±0.10	1.01±0.11	1.02±0.16	0.85±0.17	0.89±0.20	0.88±0.22	1.12±0.42
CVRi (mmHg/cm/s)†	1.57±0.31	1.55±0.36	1.63±0.43	1.60±0.37	1.25±0.17	1.24±0.15	1.33±0.17	1.34±0.14
ETCO_2_ (mmHg)	39.1±2.9	38.2±4.2	37.3±4.7	36.1±3.9	35.1±4.9	31.9±5.3	38.1±4.3	35.7±4.3
ETO_2_ (mmHg)	117.2±2.6	118.2±4.8*	116.4±5.8	120.1±4.3*	118.9±4.6	122.6±5.2*	113.9±6.5	118.5±4.0*
RR (breath/min)	16.6±1.7	17.9±2.1	16.7±1.8	16.9±2.0	18.8±4.1	19.4±4.9	17.3±4.6	17.2±3.8
Qi (L/min/m^2^)†	4.0±0.5	4.2±0.5	3.9±0.5	4.0±0.5	3.7±0.4	3.7±0.2	3.6±0.3	3.7±0.1
SVi (mL/m^2^)†	62.9±5.6	48.2±3.2*	63.3±6.4	51.8±5.1*	58.9±7.5	45.4±5.8*	55.5±4.6	45.1±5.0*
TPRi (mmHg/L/min/m^2^)	23.2±2.9	22.7±3.2	23.1±2.4	23.9±2.7	22.7±5.0	21.9±3.4	24.3±2.6	23.4±2.4

cBRS is cardiovagal baroreceptor sensitivity; SDNN is the standard deviation between R-R intervals; RMSSD is the square root of the mean squared differences between adjacent beats; pRR50 is the proportion of RR interval differences greater than 50ms divided by total beats; LF is low frequency; HF is high frequency; nu is normalized unit; SD1 is the width of a Poincaré plot; SD2 is the length of a Poincaré plot; SBP is systolic blood pressure; DBP is diastolic blood pressure; MAP is mean arterial blood pressure; CPP is cerebral perfusion pressure; MCA is middle cerebral artery; CVRi is cerebrovascular resistance index; ETCO_2_ is end-tidal carbon dioxide; ETO_2_ is end-tidal oxygen; RR is respiratory rate; Qi is cardiac output index; SVi is stroke volume index; TPRi is total peripheral resistance index

*indicates a main effect of tilt

†indicates a main effect of sex; Values are mean ± 95% Confidence Interval

## Results

Use of the Q-collar increased jugular vein cross-sectional area in both men and women at the end of inhalation (P = 0.003; η ^2^ = 0.30), and in women at the end of exhalation (P = 0.001; η ^2^ = 0.09). Use of the Q-collar did not significantly affect carotid artery cross-sectional area in men or women at the end of inhalation (P = 0.09); however, carotid area was significantly lower in both men and women at the end of exhalation (P = 0.022; η ^2^ = 0.03; Fig 1).

### Paced deep breathing and Valsalva maneuver

There were no effects of wearing the collar or sex on the heart rate responses to paced deep breathing (P>0.05; Table 1). During the Valsalva maneuver, men had higher systolic blood pressure at baseline (P<0.001; η ^2^ = 0.64), Phase 2 minimum (P = 0.006; η ^2^ = 0.38), and Phase 2 end (P = 0.005; η ^2^ = 0.43) compared to women. Men also had a larger drop in systolic blood pressure from Phase 2 end to Phase 3 (P<0.001; η ^2^ = 0.56). Wearing the Q-collar resulted in higher systolic blood pressure at Phase 2 minimum (P = 0.045; η ^2^ = 0.02) and Phase 2 end (P = 0.015; η ^2^ = 0.01) in both men and women resulting in a smaller drop from baseline to Phase 2 minimum in both sexes (P = 0.012; η ^2^ = 0.03). In women, wearing the collar increased baseline diastolic (P = 0.026; η ^2^ = 0.09) and mean brain blood flow velocity (P = 0.041; η ^2^ = 0.06) before the Valsalva maneuver whereas in men wearing the collar increased pressure recovery time during the Valsalva maneuver (P = 0.042; η ^2^ = 0.05). While men had a higher adrenergic score compared to women (P = 0.025; η ^2^ = 0.18; according to [19]), there were no effects of wearing the collar on adrenergic score or the Valsalva HR ratio (P>0.05; Table 2).

During the Valsalva maneuver systolic blood pressure decreased lower than baseline at Phase 2 min (P<0.001) and increased higher than baseline at Phase 2 end (P = 0.012), with no effect of sex or collar (η ^2^ = 0.31; Fig 2A). Similarly, men and women decreased MCA systolic flow velocity below baseline at Phase 2 minimum (P<0.001) which increased at Phase 2 end (P<0.001) yet remained below baseline (P = 0.013; η ^2^ = 0.31). There was no effect of sex or collar (P>0.05; Fig 2B). Men and women increased diastolic blood pressure at Phase 2 end compared to both baseline (P<0.001) and Phase 2 minimum (P<0.001; η ^2^ = 0.48); however, the increase was greater in men than women (P<0.001; η ^2^ = 0.04; Fig 2C). Men and women had a fall in MCA diastolic flow velocity below baseline at Phase 2 minimum (P<0.001) with an increase above baseline at Phase 2 end (P<0.001; η ^2^ = 0.59); however, the increase was attenuated in women wearing the collar at Phase 2 end (P = 0.009; η ^2^ = 0.05; Fig 2D). Men and women had an increase of mean arterial blood pressure at Phase 2 end compared to both baseline (P<0.01) and Phase 2 minimum (P<0.01; η ^2^ = 0.40), and the increase was greater in men than women (P<0.001; η ^2^ = 0.05; Fig 2E). Men and women had a reduction of MCA mean blood flow velocity at Phase 2 minimum to below baseline (P<0.001; η ^2^ = 0.68) which recovered back to baseline at Phase 2 end (P>0.05); however, at Phase 2 end women not wearing the collar had a greater increase of flow velocity compared to men not wearing the collar (P = 0.024; η ^2^ = 0.02; Fig 2F).

### Head-up tilt

The maximum HR during tilt in men was 102±8bpm without the collar and 100±8bpm while wearing the collar. The maximum HR during tilt in women was 100±10bpm without the collar and 103±12bpm while wearing the collar. There were no main effects of sex (P = 0.97) or collar (P = 0.78) with no interaction effect (P = 0.12). Men had significantly lower cBRS (P<0.001; η ^2^ = 0.26), higher systolic blood pressure (P<0.001; η ^2^ = 0.53), higher mean arterial blood pressure (P = 0.001; η ^2^ = 0.21), lower mean MCA velocity (P<0.001; η ^2^ = 0.19), higher cerebrovascular resistance index (P = 0.001; η ^2^ = 0.18), higher cardiac output index (P = 0.013; η ^2^ = 0.12), and higher stroke volume index (P = 0.002; η ^2^ = 0.08) compared to women throughout the tilt test.

In both men and women upright tilt decreased cBRS; however, the decrease was greater in women (Interaction effect P = 0.015; η ^2^ = 0.04). In both men and women, upright tilt resulted in lower cBRS (P<0.001; η ^2^ = 0.26), lower SDNN (P = 0.007; η ^2^ = 0.12), lower RMSSD (P<0.001; η ^2^ = 0.32), lower pRR50 (P<0.001; η ^2^ = 0.34), lower total power (P = 0.004; η ^2^ = 0.13), higher LF power (P<0.001; η ^2^ = 0.49), lower HF power (P<0.001; η ^2^ = 0.46), higher LF/HF (P<0.001; η ^2^ = 0.31), lower SD1 (P<0.001; η ^2^ = 0.32), lower SD2 (P = 0.022; η ^2^ = 0.09), higher HR (P<0.001; η ^2^ = 0.50), lower CPP (P<0.001; η ^2^ = 0.34), lower mean MCA velocity (P<0.001; η ^2^ = 0.19), lower systolic MCA velocity (P = 0.004; η ^2^ = 0.14), lower diastolic MCA velocity (P = 0.007; η ^2^ = 0.12), higher end-tidal O_2_ (P = 0.041; η ^2^ = 0.07), and lower stroke volume index (P<0.001; η ^2^ = 0.45). There were no effects of wearing the collar on the HRV, cerebrovascular, cardiovascular, or respiratory responses to tilt (P>0.05; Table 3).

## Discussion

In support of our first hypothesis, the Q-collar compressed both the jugular vein and carotid artery resulting in increased or decreased cross-sectional area (CSA) above the collar, respectively. The increase of jugular vein CSA was more prominent in women as it was significant at both end-inhalation and end-exhalation. During Valsalva, wearing the collar resulted in higher systolic blood pressure throughout Phase 2 in both sexes suggesting greater sympathetic/adrenergic activation. In women, wearing the collar increased diastolic and mean MCA velocity before the Valsalva (suggesting greater resting brain blood flow) resulting in a smaller increase of diastolic MCA velocity at the end of Phase 2 of the Valsalva maneuver.

Hatt et al. found that under normal circumstances the majority of cranial outflow was via the internal jugular vein (supine position), yet during jugular compression some of the cerebral fluid outflow was diverted to non-jugular pathways (i.e. increased cerebral aqueduct flow of cerebrospinal fluid)[22]. Similarly, Schreiber et al. found that when the internal jugular and deep cervical vein are compressed by circular neck compression vertebral vein flow increases [23]. However, both Hatt et al. and Schreiber et al. used a majority of male participants (6/9 and 9/12, respectively) without investigating sex differences. We found that women, but not men, exhibited an increase of supine resting diastolic and mean MCA flow velocity while wearing the Q-collar suggesting either greater inflow or impaired outflow from the brain. Favre and Serrador recently found that women who are not taking oral contraceptives display greater cerebral autoregulation compared to men [15] suggesting that reduced jugular venous drainage from wearing the collar could potentially be more readily compensated for in women. Further, Geary et al. found that male rats had both greater myogenic tone and reduced vascular distensibility in cerebral arteries during increases of pressure (such as that elicited by the reduction of venous drainage while wearing the collar) compared to females [24] perhaps contributing to the necessity of engaging secondary outflow pathways in males. We suggest that wearing the collar in women could be eliciting a greater degree of cerebral vasodilation to compensate for the increased cerebral volume via autoregulation while men could be predominately increasing outflow via non-jugular pathways such as an increase of cerebrospinal fluid flow or through increased vertebral vein flow.

Fu et al. found no sex differences in sympathetic baroreflex sensitivity during Valsalva [13]. Similarly, our group previously found no sex differences in the blood pressure response to late phase 2 of the Valsalva maneuver [14]. However, in the current study while we also found no sex differences in baroreflex sensitivity (via adrenergic score calculations), we did observe that men had a greater diastolic and mean blood pressure response to late phase 2 of the Valsalva compared to women. We suggest that these latter results in men could be influenced by the inclusion of two previously undiagnosed hypertensive men. The inclusion of hypertensive men may have exaggerated their adrenergic response since patients with hypertension have reduced baroreflex sensitivity resulting in greater sympathetic output [25, 26]. Even though there were no sex differences, in both sexes wearing the collar increased absolute systolic blood pressure at the minimum and end of Phase 2 of the Valsalva maneuver suggesting greater adrenergic activity elicited by the neck pressure. The neck pressure likely decreased the carotid transmural pressure which in turn would have increased the sympathetic and blood pressure response during Valsalva. However, this effect was not seen in the adrenergic scores (Novak or Low calculations) perhaps indicating a minimal effect of wearing the collar. In fact, an elongation of the pressure recovery time was only evident in men wearing the collar, yet this small increase is not likely physiologically relevant as it is near previously documented ranges for healthy participants (2.95±2.72s, n = 107)[27].

Contrary to our second hypothesis, there were no sex differences during upright tilt and no effect of wearing the neck compression collar despite previous findings that neck pressure in the upright posture enhances peripheral vascular responses [28]. It is possible that the differences are due to the timing and order of our testing protocols (i.e. Valsalva was always conducted first potentially influencing the responses during the subsequent upright tilt test). However, we hypothesize that the effect of the collar is limited to a less erect posture (i.e. Valsalva was conducted at only 20^o^ head-up tilt). Indeed, in the fully upright posture a greater proportion of blood drainage from the brain occurs via the structurally protected vertebral venous plexus [29], which could have attenuated any effect of jugular compression, whereas during supine Valsalva most venous drainage is via the affected internal jugular vein [29].

### Limitations

We found that during baseline measurements, prior to the Valsalva maneuver, wearing the collar increased diastolic and mean MCA velocity in women; however, this was not evident in the baseline measurements prior to tilt. These results could potentially indicate that wearing the collar dilates the cerebral vasculature of women prior to tilt thus reducing resting flow velocity back to a homeostatic level. Accurate measurements of the diameter of the MCA using magnetic resonance imaging (rather than relying on velocity measurements alone) are important to accurately determine the effect of wearing the collar on brain blood flow. Similarly, we could not obtain longitudinal images or velocity measurements in either the jugular vein or carotid artery above the collar to accurately measure cerebral inflow or outflow. This was due to the physical limitations of neck length of our participants, ultrasound probe size, and collar usage. Importantly, the Q-collar was designed to be used during exercise, yet we did not investigate its use during exercise despite plentiful evidence that exercise influences blood pressure, sympathetic outflow, and brain blood flow (reviewed in [30]). Future studies should be conducted to confirm our findings during and after bouts of exercise. Lastly, a few of our comparisons were slightly underpowered (between 0.5–0.8) and therefore we suggest that future studies include a larger sample size.

## Conclusions

We observed that the use of the Q-collar compressed both the internal jugular and the carotid artery in men and women resulting in greater adrenergic control of blood pressure during Valsalva. In women, wearing the collar also increased resting brain blood flow velocity leading to a smaller increase of brain blood flow velocity during Valsalva. There were no effects of the collar on blood pressure or brain blood flow velocity in the upright posture

## Supporting information

S1 Data Online data file. (XLSX)
